# cFinder: definition and quantification of multiple haplotypes in a mixed sample

**DOI:** 10.1186/s13104-015-1382-7

**Published:** 2015-09-07

**Authors:** Norbert Niklas, Julia Hafenscher, Agnes Barna, Karin Wiesinger, Johannes Pröll, Stephan Dreiseitl, Sandra Preuner-Stix, Peter Valent, Thomas Lion, Christian Gabriel

**Affiliations:** Red Cross Transfusion Service for Upper Austria, Krankenhausstraße 7, 4017 Linz, Austria; University of Applied Sciences Upper Austria, Softwarepark 11, 4232 Hagenberg, Austria; Children’s Cancer Research Institute, Vienna, Austria; Division of Hematology and Hemostaseology, Department of Medicine I, Ludwig Boltzmann Cluster Oncology, Medical University of Vienna, Vienna, Austria

**Keywords:** Mixed sample, Haplotype identification, Clone quantification, Next-generation sequencing, Software

## Abstract

**Background:**

Next-generation sequencing allows for determining the genetic composition of a mixed sample. For instance, when performing resistance testing for *BCR*-*ABL1* it is necessary to identify clones and define compound mutations; together with an exact quantification this may complement diagnosis and therapy decisions with additional information. Moreover, that applies not only to oncological issues but also determination of viral, bacterial or fungal infection. The efforts to retrieve multiple haplotypes (more than two) and proportion information from data with conventional software are difficult, cumbersome and demand multiple manual steps.

**Results:**

Therefore, we developed a tool called cFinder that is capable of automatic detection of haplotypes and their accurate quantification within one sample. *BCR*-*ABL1* samples containing multiple clones were used for testing and our cFinder could identify all previously found clones together with their abundance and even refine some results. Additionally, reads were simulated using GemSIM with multiple haplotypes, the detection was very close to linear (R^2^ = 0.96). Our aim is not to deduce haploblocks over statistics, but to characterize one sample’s composition precisely. As a result the cFinder reports the connections of variants (haplotypes) with their readcount and relative occurrence (percentage). Download is available at http://sourceforge.net/projects/cfinder/.

**Conclusions:**

Our cFinder is implemented in an efficient algorithm that can be run on a low-performance desktop computer. Furthermore, it considers paired-end information (if available) and is generally open for any current next-generation sequencing technology and alignment strategy. To our knowledge, this is the first software that enables researchers without extensive bioinformatic support to designate multiple haplotypes and how they constitute to a sample.

**Electronic supplementary material:**

The online version of this article (doi:10.1186/s13104-015-1382-7) contains supplementary material, which is available to authorized users.

## Background

The onset of next-generation sequencing (NGS) platforms in many laboratories enables researchers to quantify the genetic composition of a specific sample. Assuming that each cell of this sample produces the same amount of DNA a relative quantification can be deduced from the overall amount of sequencing reads [1].

454 sequencing allows for long reads up to 1000 bp, reading through most target regions where phasing is necessary. 454’s pyrosequencing strategy reads one template DNA molecule in either forward or reverse direction [2]. Illumina can also do paired end sequencing, where every DNA fragment is read in both directions and can be matched, even if there is no overlap. It is evident that both technologies allow for the detection of haplotypes although different sequencing strategies must be applied.

However, the detection of compound mutations as in *BCR*-*ABL1* for resistance testing and the quantification of the found clones have to be performed manually [3]. This process is not just time-consuming but also error prone. To our knowledge there is no software yet that can effectively detect and exactly quantify multiple haplotypes without extensive bioinformatics assistance. There are some tools (e.g. HaplotypeCaller of GATK) that can establish phasing but these tools provide no ability to detect more than two clones or calculate quantities. GS Amplicon Variant Analyzer (AVA, 454 Life Sciences, Branford, CT) is the only software that allows the definition of combined variants manually, with additional manual calculation steps. CLCbio’s Genomics Workbench 7.0 (GWB, CLCbio, Aarhus, Denmark) is able to link mutations in the same coding triplet only.

Ultra-deep sequencing with a coverage of multiple 10 k reads per target is necessary to yield accurate quantities [4]. Because exact phasing of whole genome or whole exome data is impossible and approximation just feasible statistically [5], we assume a rather small size (up to 10 k bp) as a region of interest in the human genome (trade-off between region size and coverage) [6].

cFinder is capable of analysing sequence data independent of any sequencing platform, organism and library preparation strategy. Prerequisites are a reference sequence and a medical or biological expert. Moreover, a graphical user interface provides convenient usage, so that our tool can be applied without bioinformatics or scripting knowledge. Our aim is not to sequence multiple samples and to deduce haploblocks (multiple variants passed on together by meiosis) over statistical occurrences in a population, but to characterize one sample and its composition precisely.

### Technicalities

The software can be run on any desktop computer with.NET 4.0 installed, memory usage and runtime are both reduced to a minimum. Assuring the highest degree of flexibility, no mapping or alignment algorithm is directly implemented (see Additional file 1).

Thus, the software is able to work with the most common mapping formats ACE and SAM (unpadded and padded) [7]. An annotation file (genebank format *.gb or user defined) allows the calculation of amino acid and coding sequence changes.

The architecture of the software allows for loading an enormous amount of reads (coverage), just affecting loading time. For 4 M simulated reads in paired end mode, cFinder consumed 811 MB memory and took 11.2 min to load the data, calculation was done in 11 s; for 8 M simulated reads loading took 23.8 min and calculation 22 s; conducted with 2.67 GHz and 6 GB RAM.

The output of the software is a list of connected variants (haplotypes) occurring with the same pattern on multiple reads. Additionally the coding region change and the amino acid change are reported. Hence, it can be investigated what variants occur together. The absolute number of reads with this variant combination is displayed together with the calculated percentage. This information may be exported in csv or MS Excel format.

### Usage and workflow

**Fig. 1:**
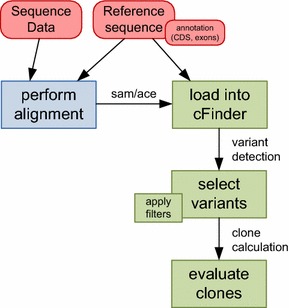
Workflow for using cFinder. Sequencing data is aligned against a reference sequence by a user defined tool (*blue*) and result is loaded in sam or ace format (together with optional annotation information) into cFinder, where variant detection is accomplished and optional filtering can be performed. After selection of desired variants clones are automatically calculated and presented for further evaluation

All tasks after the initial loading step run in linear time (target region size or coverage), the workflow is summarized in Fig. 1.

The software parses the mapping file and associates found haplotypes per read. At this step all variations (including sequencing errors) are used to create the haplotypes. Consecutive variants following each other are reported in a combined and single format, likewise are variants in one coding triplet.

Found variants are displayed and filtering is feasible. Subsequently, the user may select relevant variants. These can be designated for a specific genus of bacteria or variants that are responsible for resistance against medication [8], e.g. variant definitions from COSMIC database can be loaded.

The previously stored haplotypes are recalculated based on the selected variants and reported together with their percentage. Haplotypes as well as variants can be exported; hence, monitoring a patient at different time points is possible.

## Methods

The percentage of a haplotype is calculated with

$$percentage = \frac{haplotypeHits}{{\mathop \sum \nolimits_{i}^{variants} {\text{coverage}}(variants_{i} )}},$$

the number of reads with this haplotype’s variant composition divided by the coverage at these positions. Coverage might vary over the reference sequence. Reads not covering the variant positions will be counted as wild type (wt), hence the sum of all percentages will not sum up to 100 % and wt percentage is estimated from detected clones ($1 - \mathop \sum \nolimits percentages$, minimum 0).

Only sequence reads exactly matching the variants of the assigned haplotype are counted. There is no further statistics applied to the found haplotypes, only the extraction of all occurrences in a highly efficient way. Forward and reverse fragments of paired-end reads (if available) are connected to establish a haplotype. Ambiguous bases in a paired-end overlap are not counted as a variant (by definition one of them is a sequencing error).

### Overlapping amplicons

**Fig. 2:**
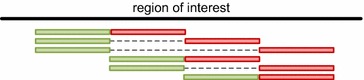
Overlapping multi-amplicon paired-end design. Schematic representation of covering a region larger than the maximum reading length, haplotypes are therefore scattered in multiple sequence reads. Reference sequence is displayed in *black*, amplicons in forward (*green*) and reverse (*red*), pairs (but not covered regions) are denoted with a *dashed line*

Specialized amplicon designs have to be developed when read length is shorter than region of interest (see Fig. 2). If this is not uniformly covered by design, the amplicon positions must be loaded (to perform correct calculations). For these amplicon designs the user finds a specialized checkbox “Infer Relationship(s)” where haplotypes scattered over multiple amplicons are again reconnected. The algorithm creates a symmetric, unweighted graph with nodes representing variants and edges representing an occurrence on (at least) one sequence. In this graph, the largest subgraph is determined where all nodes are connected with all other nodes in the subgraph (finding this graph is referred to as maximum clique problem in graph theory). The subgraph defines a new haplotype, and readcounts for contributing fragments are recalculated. Fragments and connections with no reads left are removed and the next largest subgraph is searched. Since the maximum clique problem is NP-complete [9], this is the only computationally expensive task after loading. It is limited to 15 rounds and 20 variants to be accomplishable on a desktop computer. The output for an overlapping amplicon design does not differ from the above described output. Concerning the connections between variants, some might be detected that cannot be found on reads. It should be noticed that this combinatorial task is complex and sophisticated and there are cases where no connection can be achieved at all, especially with high number of variants and overlapping subgraphs.

## Results and discussion

Next-generation sequencing data generated from long-range *BCR*-*ABL1* sequencing was utilized for evaluation and testing [8]. The original data consist of sequencing one 933 bp amplicon for eleven samples with 29 clones detected by manual investigation (using 454 GS FLX+ technology, AVA, CLC GWB and manual calculation of clones and percentages), ten samples were used for subsequent analysis (see also Additional file 2). Approval of an ethics committee is not required for this analysis. Alignments of individual reads from AVA and CLC GWB using the Large Gap Mapper tool (settings m2, i3, d1, s0.7, l0.7) were loaded and annotated in our cFinder, and both compared to previous (manual) results from [8]. Clinically relevant variants were selected and clone detection yielded 38 clones in 10 samples. All previously detected clones could be confirmed and clones with a percentage greater or equal to 1 % were investigated. Correlations of clones’ percentages with manual data were 0.973 for AVA as well as for CLC alignments and correlations of counted number of reads were 0.995 and 0.993 for AVA and CLC, respectively.

Since alignment of a large 540 bp deletion (well-known for the BCR-ABL fusion product) in sample 8 is not possible by AVA, it was just analyzed with the alignment created by CLC. All clones with a percentage greater or equal to 1 % were investigated.

A detailed comparison is presented in detail in Table 1. For four samples more clones were detected, representing sub clones of the manually defined ones. In one of them, this leads to reduced percentages of previously reported clones. Just five clones differed by more than 10 percentage points, although a similar number of reads was reported (different calculation).

**Table 1:** Comparison of alignment methods and cFinder to manual approach

Sample (no.)	Clone variants	Manual		cFinder (AVA)		cFinder (CLCbio)	
		%	Hits	%	Hits	%	Hits
1	c.749 G>A	22.0	14,956	23.3	15,611	22.43	14,745
	c.757 T>C	26.5	18,458	30.6	20,886	29.39	19,604
2	c.749G>A, c.943A>G, c.1497A>G	15.7	4748	6.4	4362	6.21	4066
	c.749G>A, c.949T>A, c.1497A>G	4.1	1240	2.4	1641	2.3	1522
	c.749G>A, c.1497A>G	22.7	6864	7.1*	4145	6.7*	3729
	c.943A>G, c.1497A>G	17.1	16,237	12.6	9183	12.5	8715
	c. 949T>A, c.1497A>G	3.3	3128	4.5	3342	4.6	3240
	c.1497A>G	45.2	26,522	61.2*	35,867*	61.3*	33,804
	c.749G>A, c.949T>A	–	–	3.4	2519	3.5	2488
	c.749G>A	–	–	13.9	7996	14.2	8074
	c.749G>A, c.943A>G	–	–	10.8	7838	10.9	7692
	c.943A>G	–	–	4.0	3483	4.2	3574
	c.949T>A	–	–	1.1	991	1.2	1023
3	c.1375G>A, c.1423_1424ins35	6.2	3495	5.9	3186	6.4	3019
	c.1375G>A	90.9	51,247	90.9	51,891	87.9	42,693*
4	c.730A>G	20.3	9887	27.5	17,769	27.5	17,483*
5	c.756G>T, c.1086_1270del185	5.4	1407	6.2	1406	6.8	1490
	c.756G>T, c.1423_1424ins35	1.0	160	1.6	244	0.2**	30
	c.756G>T	41.0	10,845	58.9*	12,446*	57.3*	11,545
	c.1423_1424ins35	–	–	1.6	244	1.6	235
	c.1086_1270del185	2.7	727	2.6	625	2.6	611
	c.888_919del32	1.6	427	0.9**	220	0.2**	40
6	c.756G>>T, c.1086_1270del185	20.4	8151	14.1	7922	14.1	7702
	c.1086_1270del185	–	–	6.3	4008	4.6	2828
	c.756G>T	64.6	32,472	65.0	31,932	63.3	30,093
7	c.756G>T	51.2	38,883	62.3*	38,726	61.5*	37,336
8	c.838_1378del540, c.1423_1424ins35	98.4	49,425	–	–	85.7*	47,729
	c.1423_1424ins35	–	–	–	–	2.1	1187
	c.838_1378del540	–	–	–	–	10.8	6013
9	c.825G>A	2.2	229	3.1	269	3.0	250
10	c.1086_1270del185, c.1423_1424ins35	1.3	278	0.5**	132	0.5**	116
	c.944C>T, c.1086_1270del185	9.1	2260	7.5	2215	5.3	1548
	c.944C>T	30.6	9778	33.7	10,069	34.3	10,061
	c.1086_1270del185	12.1	3278	10.4	3026	7.4	2164
	c.1423_1424ins35	2.1	460	1.9	384	1.8	375

Sample and found clone variants are listed with their percentage of occurrence and the hits (absolute number of reads with that variant), comparing manual detection with automated analysis with cFinder with two different alignment software products. Results marked with one star (*) show intense deviation from manual findings, numbers marked with two stars (**) fell below threshold of 1 %. If clone was not detected it is marked with a dash (–)

Two clones fell below the 1 % limit with the improved calculation and would not be treated as real clones. The results generated with AVA and CLC differ for one clone only.

Deviation from manual results is evident due to different definition of compound mutations and calculation ($n = 2^{numVariants}$ possible combinations, where numVariants is the number of variants of interest). Manual calculation did not take coverage at variant position into account, while AVA does not output the number of reads concerning sub clones accurately. Our software tool detects occurring haplotypes automatically, counting each read just once and calculates the frequencies adjusted to the coverage at the variants position.

**Fig. 3:**
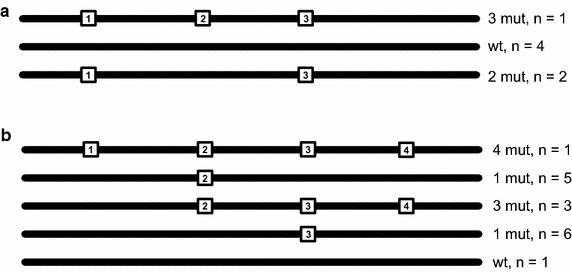
Test scenarios for overlapping amplicon design. Two test scenarios were created with simulated reads, consisting of three clones (**a**) and five clones (**b**). *Each line* represents a clone having different variants (*numbered squares*), scattered over multiple sections of amplicon design (see Fig. 2)

No real data were available for a multi-amplicon design, therefore two artificial alignments were created (Fig. 3). Our software managed to infer the haplotypes scattered over multiple amplicons and extract their correct clones and counts (Table 2).

**Table 2:** Test of simulated data with overlapping amplicons

Test	Clone variants	Default		Infer relationships	
		%	Hits	%	Hits
A	1	23.8	5	–	–
	3	23.8	5	–	–
	1, 3*	14.3	3	28.6	2
	1, 2	4.8	1	–	–
	2	4.8	1	–	–
	2, 3	4.8	1	–	–
	1, 2, 3*	–	–	14.3	1
B	3*	43.8	21	37.5	6
	2*	37.5	18	31.3	5
	2, 3	8.3	4	–	–
	3, 4	8.3	4	–	–
	2, 4	8.3	4	–	–
	4	6.3	3	–	–
	1, 4	2.1	1	–	–
	1, 3	2.1	1	–	–
	1, 2	2.1	1	–	–
	2, 3, 4*	–	–	18.8	3
	1, 2, 3, 4*	–	–	6.3	1

Comparison of using the infer relationship option to detect haplotypes that are scattered over multiple amplicons. The column clone variants holds a list of variants (numbered according to Fig. 3, design of the test cases) where “1, 3” means that the clone has variant 1 and variant 3. Default analysis yields just occurring haplotypes on simple amplicons while ticking the checkbox for inferring haplotypes manages to identify the connected variants. If clone was not detected it is marked with a dash (–), true haplotypes (used for simulation) are marked with a star (*)

**Fig. 4:**
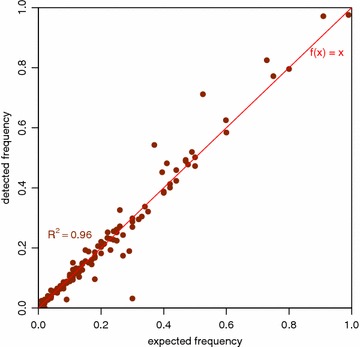
Scatterplot of simulated reads. The frequencies of clones were plotted against the actual detected frequencies. *One dot* represents one clone, perfect matches are on the *red line*

Besides, we used GemSIM to simulate 100 k reads (l = 600) for a short (924 bp) fragment of *E. coli* using the profile for 454 data [10]. In total 26 test cases had 133 different haplotypes (min. 3, max. 8 haplotypes with each up to 9 variants, avg. 4.2 variants). We included multiple clones (haplotypes) that were created by the GemSIM software (GemHaps.py) along with sequencing errors. After alignment with CLC GWB the cFinder detected all haplotypes. Using the absolute number of reads detected the estimated percentages correlated with R^2^ = 0.96 that is nearly linear. The deviation can be related to the high number of errors included in the 454 profile. Figure 4 displays a scatterplot visualizing the simulated and detected percentages.

## Conclusion

cFinder is easy to use and quickly identifies and quantifies clones in a mixed sequencing result this discriminates the cFinder from other software products. Overall, all clones and their frequencies are designated with a minimum of user interaction. The tool can be used for data analysis for research as published in [8]. Both alignment types (AVA and CLC) showed a similar performance, supported the previous manual findings, and could even refine them in some cases. The simulation experiment confirms the high accuracy. Thus, our cFinder is completely independent from any alignment tool or settings, allowing for a maximum of flexibility for a specific application.

## Availability and requirements

- Project name: cFinder.
- Availability (including test data): http://sourceforge.net/projects/cfinder/.
- Operating system(s): Windows.
- Programming language: C#.
- Other requirements: .NET 4.0 or higher.
- License: GNU GPL v3.0
- Any restrictions for commercial usage: license needed.

## Additional files

Additional file 1:
**Archive containing the cFinder software.** Includes executable, library and settings files. Additional file 2:
**Archive containing test data.** Include annotations and exemplary alignment files from AVA and CLC for sample 5, as well as simulated test reads for overlapping amplicons.

## Abbreviations

NGS: next-generation sequencing
DNA: deoxyribonucleic acid
AVA: GS Amplicon Variant Analyzer
GS: genome sequencer
GWB: genomics workbench
COSMIC: catalogue of somatic mutations in cancer
SAM: sequence alignment/map format
