# Supplementary material for: cFinder: definition and quantification of multiple haplotypes in a mixed sample
Source: BMC Res Notes. 2015 Sep 7;8:422. doi: 10.1186/s13104-015-1382-7 (PMC4562109; doi:10.1186/s13104-015-1382-7)
Supplement: Additional file 1: — Archive containing the cFinder software. Includes executable, library and settings files. [file 13104_2015_1382_MOESM1_ESM.zip › cFinder/manual.pdf]

Austrian Red Cross, Blood Transfusion Service For Upper Austria

Manual cFinder

Inhalt

Graphical User Interface (GUI) 3
Input 5
Calculation of mutations in comparison with a reference sequence 5
Sequences file .... **Fehler! Textmarke nicht definiert.**
Annotation File 6
Output 8
Mutations/Variants 8
Filters 8
Sequence Clone Calculation 9
Menu - Data 11
Load 11
VARIANTS 11
COSMIC FILE 11
USER-DEFINED VARIANTS FILE 12
CLC EXPORTED FILE 15
AMPLICON DESIGN 15
Export 16
Export variants 16
Export clones 16
Reset 17
Close 17
Reset automatically 18
Annotation File 18
Sequences File 18
Logger 19
Features 22
Menu: GUI - Change color 22
Change window size 23

Graphical User Interface (GUI)

This software enables the detection of mutations in tumor samples (e.g. in *BCR-ABL*), the identification of clones and their haplotypes. Main goals of the application were usability and efficiency concerning runtime and memory usage.

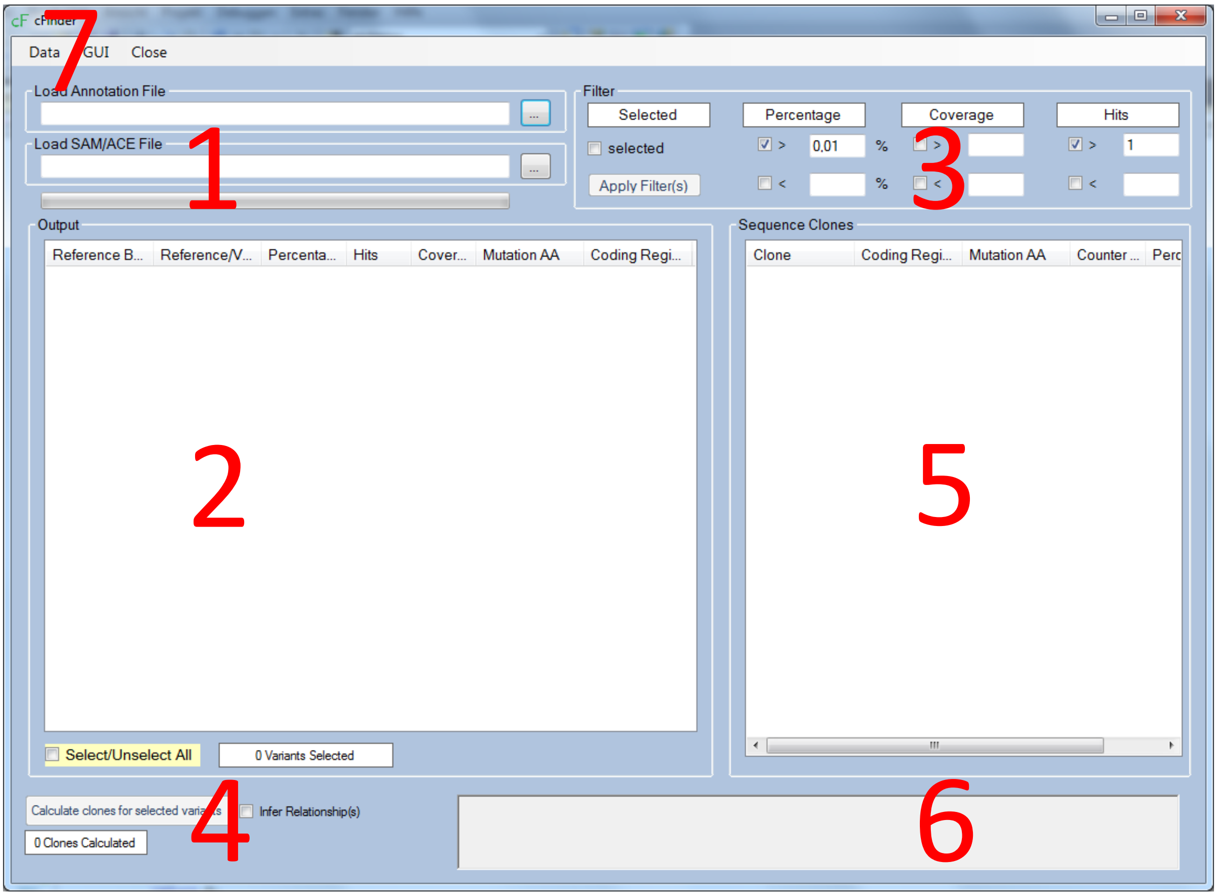

cFinder

GUI description

1. Input file section for annotation and sequences input file.
2. Variants output window. This window will contain a list of detected mutations.
3. Filter for variants output window.
4. After selection of variants by checkboxes sequence clone calculation is started with the button “Calculate clones for selected variants”. The number of calculated clones is then shown in the label below the button. The checkbox “Infer relationship(s)” may be ticked, if the user wants so identify dependencies in an overlapping amplicon design.
5. In this output window found sequence clones are displayed.
6. Window logs tasks and outputs current status and important messages.
7. The menu section is important for loading or exporting different files and for resetting data and/or annotation information.

Standard workflow

1. The user loads an annotation file including information about the start of the coding sequence (CDS) and exon ranges. Optional step if annotation (amino acid change) is desired.

2. The user loads a SAM/ACE alignment file. With this file the mutations (= variants) are detected and subsequently annotated.
   For further information about the input files (setting etc.), please take a look at section "*Input - Calculation of mutations in comparison with a reference sequence*".
   The progress bar (below the input file section) shows the process of loading the alignment file and of variant detection.
   Information that will be provided for each variant:
   1. The reference position[1] at which the variant occurs.
   2. The reference base[2] at that position followed by a "/" and the variant base.
   3. The percentage of the occurrence of this variant at the specific position.
   4. Absolute number of occurrences of this variant in reads (hits).
   5. The coverage at this position (absolute number of reads at that position, regardless of the base).
   6. The amino acid change that is caused by the mutation:
      "p." for "protein", followed by the position and the amino acid change.
   7. The variant annotated to the coding sequence as nucleotide change, nomenclature according to Human Genome Variation Society. For example, c.1073delA:
      The "c." for "coding", then the position in the coding sequence and the type of change as well as the variant itself is printed out. If variant is outside coding region, this field is empty.

3. The loaded variants can be restricted by filters. The user can show just selected variants (ticked checkboxes), filter by percentage, coverage or the number of hits.
4. For clone calculation, it is necessary to select variants that denominate a clone[3] that are further used in the calculation process. The user can select variants by ticking their checkboxes or load additional files where checkboxes are ticked automatically. The clones are then identified by pushing the "Calculate clones for selected variants" button. Below this button there is a label that shows the number of different sequence clones.
5. The clones that were calculated are then presented in the output window:
   1. The clone itself listing all variants that denote this clone.
   2. The nucleotide (coding region) change of the clone.
   3. The AA change of the clone including the change of the amino acid of each variant that is included in the clone's calculation.
   4. The counter of the clone's occurrences in the loaded data set.
   5. The percentage of appearance.
6. All user activitys and errors are reported by a logger.
7. The menu provides actions for loading additional files, exporting data or resetting.

[1] The reference position refers to the position of the variant in the reference sequence without including gaps.
[2] Here, the reference base can be a gap in case of an insertion, or the variant can be a gap in case of a deletion.
[3] e.g. clinically relevant mutations

Input

Detection of variants

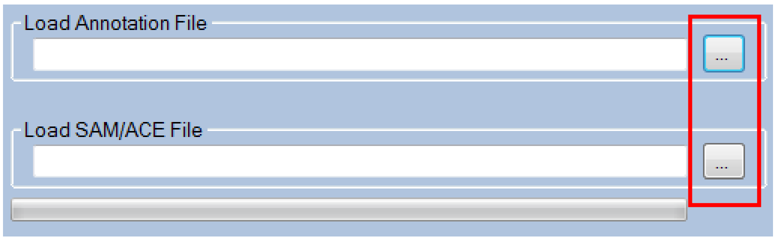

Input File Selection

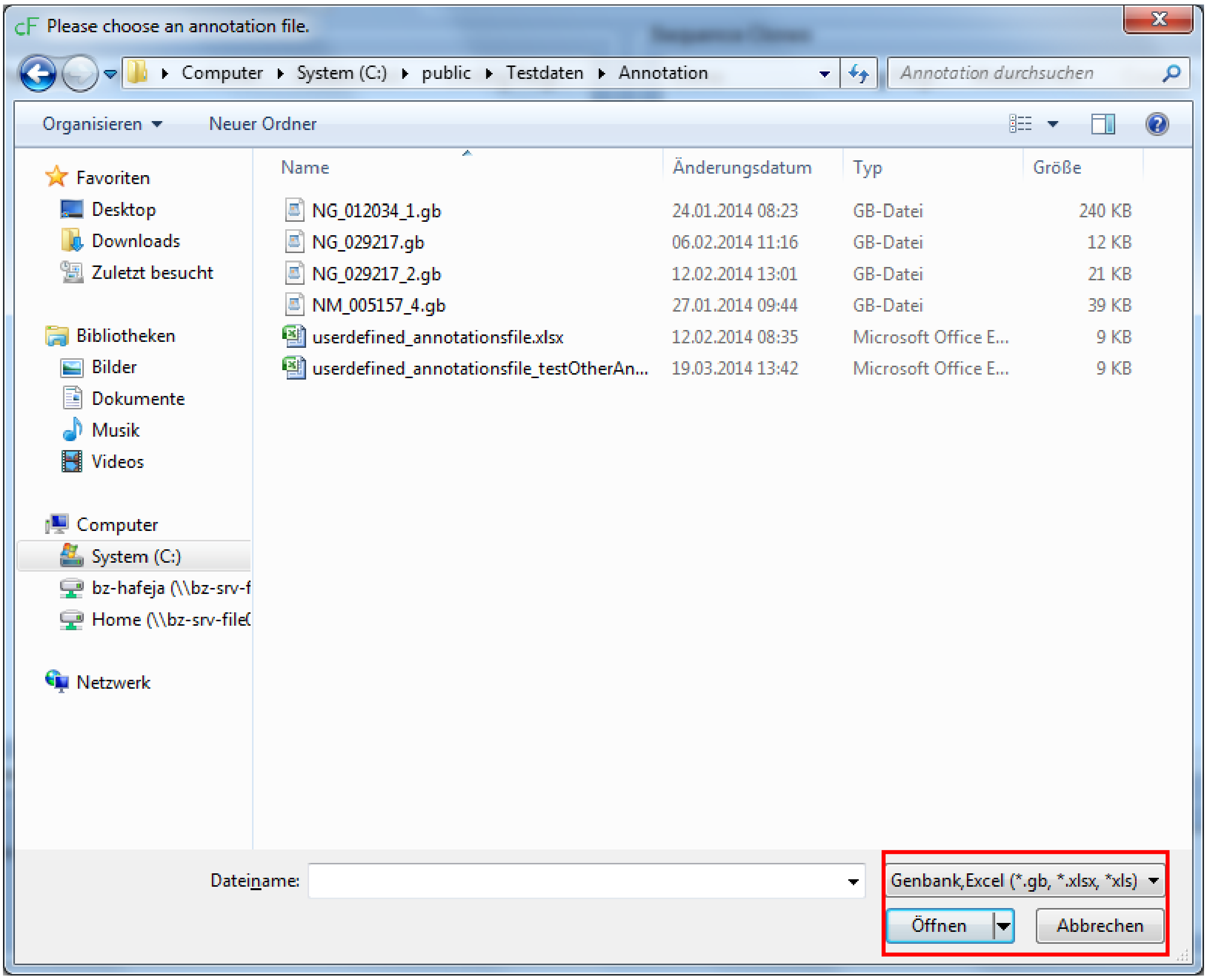

Example of the file dialog to import a file for annotation.

By clicking one of the two buttons (Input File Selection, red mark at first input screenshot), a dialog allows to choose sequence and annotation input file.

Just files in appropriate formats are displayed in the file dialog.

Annotation File

The requirements for the annotation input file are as follows:

1. The file must be a Genbank (.gb) or a user-defined Excel file (.xlsx, xls).
2. The user has to keep in mind which alignment file was/will be loaded. If the file is an unpadded SAM (does not include reference sequence), a Genbank file has to be loaded first, including a reference sequence (origin).
3. If a sequence file was/will be loaded including a reference sequence (padded SAM or ACE): If a **Genbank** file is selected, the user has to keep in mind that the Genbank origin has to be the same as the reference sequence in the sequence file. Otherwise exon borders, CDS and annotations will be wrong. In cases where reference sequence was changed by the user, an Excel file with all information has to be prepared.
4. If the user wants to **define an Excel file** by himself: Information about the start index of the coding sequence and all exons including the borders of each one (start and end index) have to be provided. CAUTION: Indexing starts at 1 (not 0). The positions of CDS start and exons have to be integers (no decimal numbers). Please note that the rows must be denoted by the same identifier as in the following example input. The start of the CDS and the exon borders must be specified in relation to the loaded reference sequence. As you see in the example below, borders can also be declared using negative integers, if an exon starts before the actual start of the reference sequence.

	A	B	C
1	Start	1	
2	exon1	-2	79
3	exon2	80	253
4	exon3	254	549
5	exon4	550	822
6	exon5	823	1053
7	exon6	1054	907
8	exon7	908	1085
9	exon8	1086	1270
10	exon9	1271	1423
11	exon10	1424	1513
12	exon11	1514	1678
13	exon12	1679	5385

User Defined Excel Annotation Input File

Sequences file

The sequences input file requirements are as follows:

1. The file must be in SAM (.sam) or ACE (.ace) format. A SAM file is a tab-delimited text file that contains information for a sequence alignment. An ACE file contains information about sequence reads (e.g. for a sequence alignment), like offset, clipping[4] starts and endings, read names, or the sequence of bases for the reads. BAM[5] (.bam) file formats are not supported, but can be converted with samtools to a SAM file by the user.

[4] Clipping is the removing of low-quality parts of a sequence at the start and end for an alignment.

Note for users of CLCbio and/or GS Amplicon Variant Analyzer:
If CLCbio is used for calculation, the user can load a SAM or ACE file.
If the user uses the 454 GS Amplicon Variant Analyzer for project setting, only SAM files can be loaded because in ACE files part of the reference sequence is missing.

During parsing just needed information is extracted and kept in memory, file is neither duplicated nor stored elsewhere.The progress bar will provide user feedback about the process.

[5] The BAM file is the binary version of a SAM file.

Output

Mutations/Variants

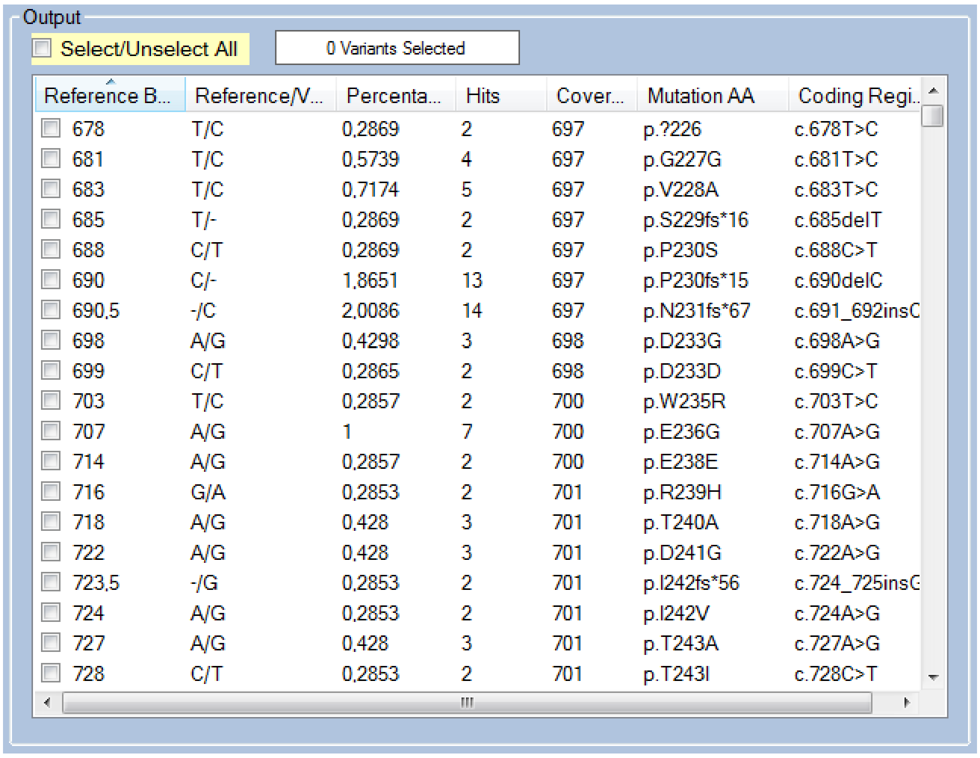

Reference B...	Reference/V...	Percenta...	Hits	Cover...	Mutation AA	Coding Regi...
678	T/C	0,2869	2	697	p.?226	c.678T>C
681	T/C	0,5739	4	697	p.G227G	c.681T>C
683	T/C	0,7174	5	697	p.V228A	c.683T>C
685	T/-	0,2869	2	697	p.S229fs*16	c.685delT
688	C/T	0,2869	2	697	p.P230S	c.688C>T
690	C/-	1,8651	13	697	p.P230fs*15	c.690delC
690,5	-/C	2,0086	14	697	p.N231fs*67	c.691_692insC
698	A/G	0,4298	3	698	p.D233G	c.698A>G
699	C/T	0,2865	2	698	p.D233D	c.699C>T
703	T/C	0,2857	2	700	p.W235R	c.703T>C
707	A/G	1	7	700	p.E236G	c.707A>G
714	A/G	0,2857	2	700	p.E238E	c.714A>G
716	G/A	0,2853	2	701	p.R239H	c.716G>A
718	A/G	0,428	3	701	p.T240A	c.718A>G
722	A/G	0,428	3	701	p.D241G	c.722A>G
723,5	-/G	0,2853	2	701	p.I242fs*56	c.724_725insG
724	A/G	0,2853	2	701	p.I242V	c.724A>G
727	A/G	0,428	3	701	p.T243A	c.727A>G
728	C/T	0,2853	2	701	p.T243I	c.728C>T

Output Window:
Reference Base Position | Reference Base/Variant | Percentage | Number of Hits | Coverage at this position | Amino Acid Change | Coding Region Change

Filters

This output of variants can be restricted by filters:

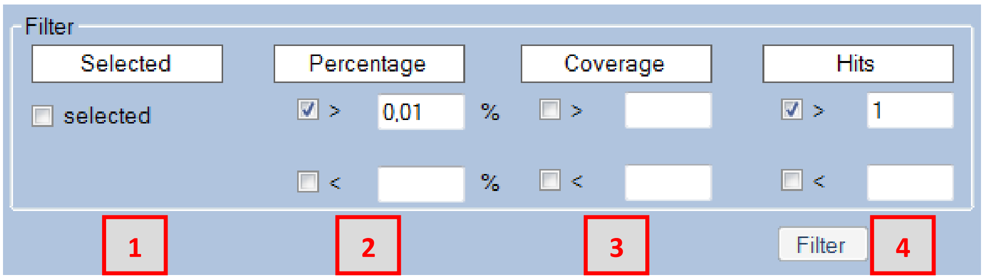

For applying any filters, the corresponding checkbox(es) must be ticked, the value inserted and then the “Apply Filter(s)” button pressed. The output window is then updated.
CAUTION: It is mandatory to tick the checkboxes of the filter to be used.

1. Filtering of *selected* variants
   It is possible to select variants by checking their checkboxes in the output window. Then the user can check the “selected” checkbox in the filter section, hence only the selected variants

are shown. This might be useful, if many variants are displayed via the output window and just a few variants on very different positions are selected, enhances overview.

2. Filtering by *percentage*
   The variants can be filtered by their percentage of appearance. The user can specify if variants should be shown with a smaller or higher percentage than a set limit (e.g. at the screenshot of this section, variants are included with a higher percentage than 0.01). The user is allowed to enter the limiting percentage as a decimal number with a comma or point as separator. Both representations will be read and processed correctly.
3. Filtering by *coverage*
   With this filter the variants are filtered by the coverage at the specific reference position. The user can specify what range of coverage is reasonable.
4. Filtering by number of *hits*
   The variants are filtered by the number of their hits. The absolute number of sequences where a specific variant occurs is called hits. The user can display variants with hits smaller or higher than the limits.

DEFAULT SETTINGS

Each time when a user loads a sequence input file, filters are set automatically and just variants with a higher percentage than 0.01 % and with more than 1 hit (hits > 1) are displayed. Each variant that is subjacent is sorted out (very likely to be a sequencing error). If desired, filter can be unset.

Sequence Clone Calculation

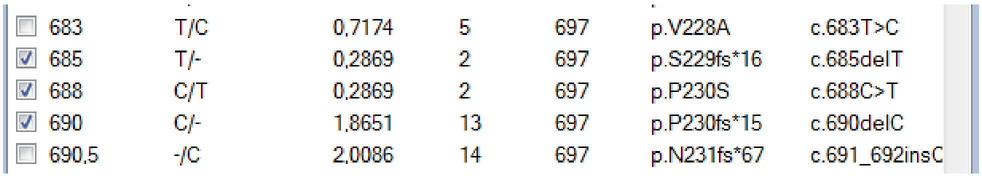

☐	683	T/C	0,7174	5	697	p.V228A	c.683T>C
☑	685	T/-	0,2869	2	697	p.S229fs*16	c.685delT
☑	688	C/T	0,2869	2	697	p.P230S	c.688C>T
☑	690	C/-	1,8651	13	697	p.P230fs*15	c.690delC
☐	690,5	-/C	2,0086	14	697	p.N231fs*67	c.691_692insC

Select variants in output window

For the calculation of (sequence) clones the user first has to select variants that should be included in the calculation by ticking their checkboxes.

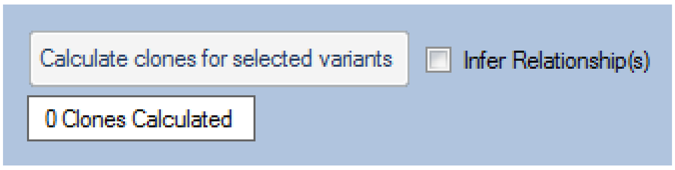

If the desired region of interest is not span by a single amplicon but multiple (overlapping) amplicons are needed to resolve the haplotype, it is necessary to tick the checkbox next to “Infer Relationship(s)”. Thus evidence is collected about variants that are not on the same read but co-occuring with others and therefore connected to an haplotype.
By clicking “Calculate clones for selected variants” button the calculation for clones starts.

Number of calculated clones is shown in the white label below the button. If the “Infer Relationship(s)” checkbox is ticked, the progress can take a while and there is user feedback in the progress bar.

If a clone is carrying more than one mutation, the output information is divided by a pipe symbol (“ | “) to have a clearer legibility.

Sequence Clones

Clone	Coding Regi...	Mutation AA	Counter Clone	Percentage
A/- | -/G | -/G	c.1073delA | ...	p.N358fs*14 |...	15	2,0633
-/C | A/-	c.887_888ins...	p.P296fs*2 | ...	102	11,8674
A/-	c.1073delA	p.N358fs*14	73	7,8834
			174	0.0
-/G	c.1193_1194i...	p.G398fs*47	13	2,0701
G/- | A/-	c.752delG | c....	p.G251fs*16 |...	13	1,5971
G/-	c.752delG	p.G251fs*16	39	5,5556
A/- | -/G	c.1073delA | ...	p.N358fs*14 |...	29	3,7323
-/C	c.887_888insC	p.P296fs*2	49	6,1791
G/- | -/G	c.752delG | c....	p.G251fs*16 |...	3	0,4511
G/- | -/C | A/- | -...	c.752delG | c....	p.G251fs*16 |...	15	2,0403
G/- | -/C | C/- | ...	c.752delG | c....	p.G251fs*16 |...	5	0,6231
G/- | -/C | -/G	c.752delG | c....	p.G251fs*16 |...	11	1,5544
G/- | -/C | -/G	c.752delG | c....	p.G251fs*16 |...	2	0,2828
-/C | C/- | -/G	c.887_888ins...	p.P296fs*2 | ...	2	0,2715
-/C | C/- | A/-	c.887_888ins...	p.P296fs*2 | ...	5	0,5981
-/C | A/- | -/G | -...	c.887_888ins...	p.P296fs*2 | ...	21	2,8245
-/G | -/G	c.1193_1194i...	p.G398fs*47 |...	10	1,5936
G/- | -/G | -/G	c.752delG | c....	p.G251fs*16 |...	10	1,533
-/C | C/-	c.887_888ins...	p.P296fs*2 | ...	2	0,2528
G/- | A/- | -/G	c.752delG | c....	p.G251fs*16 |...	5	0,6649

* estimated number

Output window sequence clones

Menu - Data

Load

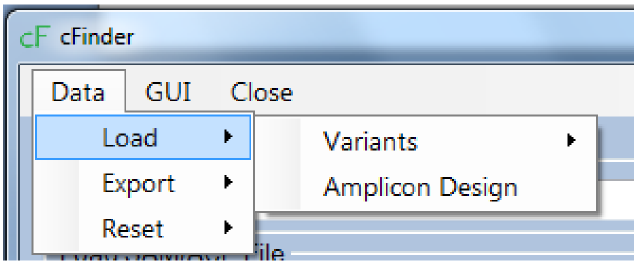

Load from existing file

The user can load variant data for selection of variants from an existing file (Cosmic file, self-defined variant file). Beforehand variants have already to be present in the output window. All variants in that file that correspond to the detected variants are then checked (additionally to already selected variants). Afterwards, the user can filter the selected variants to display them in a compressed way or to calculate sequence clones with them.
The additionally loaded input files can be:

VARIANTS

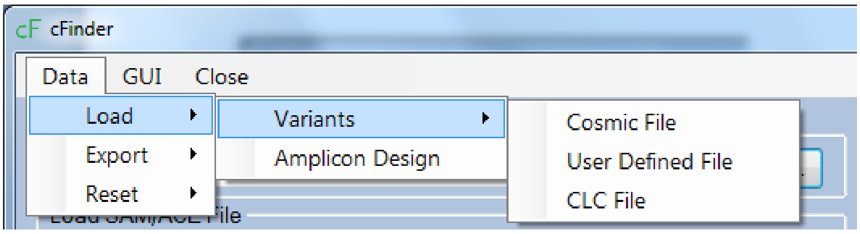

COSMIC FILE

For using cosmic variants, the annotation file must already be loaded. The content of second column (“`CDS Mutation`”) in the cosmic file is the most important and should not be changed. It is mandatory to have annotation information because the coding DNA information is compared with the ones in the cosmic file.

Where to find such a file?
It is possible to download a file containing mutations from the COSMIC website
http://cancer.sanger.ac.uk/cancergenome/projects/cosmic/

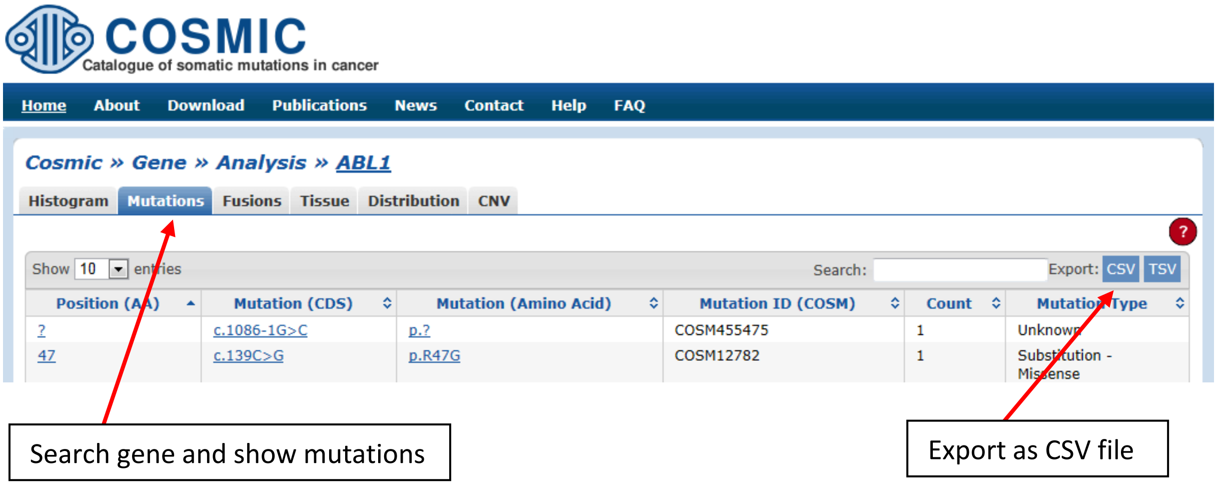

How the downloaded file could look:

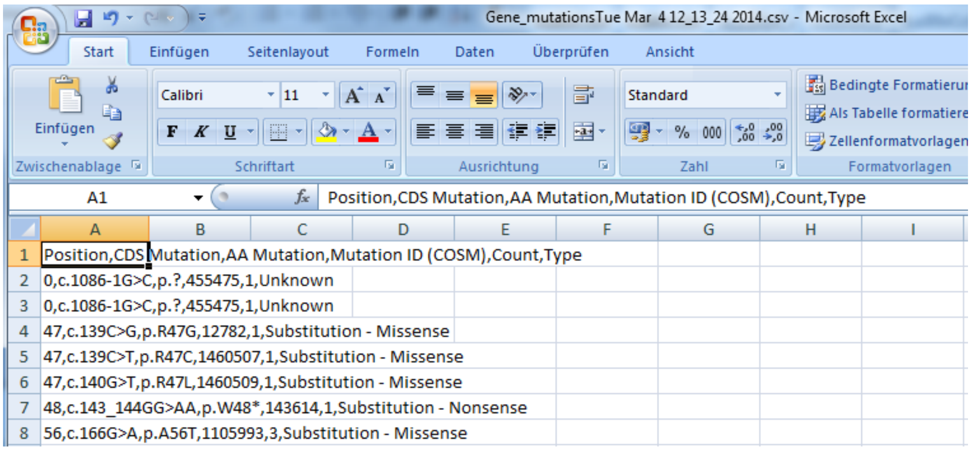

COSMIC CSV: comma separated information about position, mutations, count and types

USER-DEFINED VARIANTS FILE

The user can define a variants file. The mandatory headers have to be named exactly like the headers in the variants output window:

```
Reference Base
Reference/Variant
Percentage
Hits
Coverage
Mutation AA
Coding Region Change
```

Columns can be in any order and not all of them have to be present. Important for checking the right variants are "**Reference Base**", "**Reference/Variant**", "**Mutation AA**" and "**Clone Variants**", at least one of them has to be present for right calculation. The other columns will be ignored.

Depending which information is used as input, there are following **possibilities**:

Case "**Hits**"/"**Percentage**"/"**Coverage**":

These columns are not used for selecting variants because this information depends on the actual sample. If only these columns are given, there will be no variant checked.

	A	B	C
1	Percentage	Hits	Coverage
2	0,2437	3	1230,8214
3	0,066	3	4546,3333
4	0,11	5	4546,3333
5	0,0974	13	13347

no variants can be selected

Different variations of columns

In this section, either one of the 4 important columns (“**Reference Base**”, “**Reference/Variant**”, “**Mutation AA**” and “**Coding Region Change**”) has to be present for variant selection. *The more of these 4 columns are given, the more stringent are the results of variant selection.*

Case “**Reference Base**”, “**Reference/Variant**”, “**Mutation AA**” and “**Coding Region Change**”:

If all 4 columns exist in the file, all 4 columns are used to check the variants in the variants output window. So the entries of all columns have to correspond to the variant (a loaded annotation file is mandatory).

	A	B	C	D	E	F	G	H
1	Reference Ba	Reference/V	Percentage	Hits	Coverage	Mutation AA	Coding Region Change	
2	678	T/C	0,2869	2	697	p.?226	c.678T>C	
3	681	T/C	0,5739	4	697	p.G227G	c.681T>C	
4	683	T/C	0,7174	5	697	p.V228A	c.683T>C	
5	685	T/-	0,2869	2	697	p.S229fs*16	c.685delT	

example file in excel format

	A	B	C	D	E	F	G
1	Percentage	Hits	Reference B	Mutation AA	Reference/V	Coverage	Coding Region Change
2	0,2437	3	677	p.?GVSPNYD	AGTCCAAAA(	1230,8214	c.677_704subst28
3	0,066	3	677	p.?226fs*69	AGTCC-/GGT(	4546,3333	c.677_678ins1
4	0,11	5	677	p.?226fs*271	AGTCCC/GGT	4546,3333	c.677_682del1
5	0,0974	13	677	p.?226	A/G	13347	c.677A>G

all columns

```
Reference Base;Reference/Variant;Percentage;Hits;Coverage;Mutation AA;Clone Variants
677;A/G;0,0974;13;13347;p.?226;c.677A>G
677;AGTCCC/GGTCC-;0,11;5;4546,3333;p.?226fs*271;c.677_682del1
677;AGTCC-/GGTCCC;0,066;3;4546,3333;p.?226fs*69;c.677_678ins1
677,5;-T/AC;0,0299;4;13357;p.?226fs*72;c.677_678ins1
677,5;-/A;8,6312;1152;13347;p.?226fs*72;c.677_678insA
678;T/G;0,015;2;13367;p.?226;c.678T>G
```

different column order, file in csv format

Case **3 columns of 4**:

There are 3 columns in the input file given. These columns are used to check the variants in the variants output window. So the entries of these columns have to correspond to the entry in the output window. The column missing is not used for selection.

Case **2 columns of 4**:

If 2 columns are given, only these two are used to check the variants in the variants output window. So the entries of both columns have to correspond to the variant.

	A	B	C	D	E
1	Percentage	Hits	Reference Base	Reference/Variant	Coverage
2	0,2437	3	677	AGTCCAAAAGATAC	1230,8214
3	0,066	3	677	AGTCC-/GGTCCC	4546,3333
4	0,11	5	677	AGTCCC/GGTCC-	4546,3333
5	0,0974	13	677	A/G	13347

example: variants will be selected by Reference Base Position and Reference/Variant column; no annotation needed

Case **1 column of 4**:

There is just one column used for selection of the right variants. So this input file would return a wide variety of variants.

	A	B	C	D
1	Percentage	Hits	Reference Ba	Coverage
2	0,2437	3	677	1230,8214
3	0,066	3	677	4546,3333
4	0,11	5	677	4546,3333
5	0,0974	13	677	13347

variants will just be selected depending on Reference Base Position; no annotation needed

File formats can be Excel (.xls, xlsx), Text (.txt) or CSV (.csv).

CAUTION: Each column and value in a **text file** has to be divided by a ';' or a ','. If a comma is used for separating the columns, the user must use a point as decimal separator![6]

CAUTION: An user may load a CSV variants file that was exported by menu selection "Export – Variants" (section "*Menu – Export – Export variants*"). If the user reloads a variant file he has previously exported, he has to take care that the current loaded sample file is annotated correctly according to the previous annotation. Otherwise the variants cannot be selected correctly.

As soon as the button "Calculate clones for selected variants" is clickable, the process of selecting variants has finished.

[6] Using semicolon as column separator allows the user to enter comma delimited decimal numbers.

CLC EXPORTED FILE

This file must be in Excel (.xlsx, .xls), tab-delimited text (.txt) or CSV (.csv) format. The columns of the text and CSV file have to be separated by semicolon. If the user opens (must not be resaved!) an exported CSV file with a simple editor, he will see that the columns are limited with quotation marks. These marks must not be deleted! One of the columns must be named `Coding region change`. The Ensembl Transcript ID (ENST) and the coding region change have to be given. These data has to be divided by a colon (:). A row can contain more changes than one, but they have to be separated by semicolon (see example beneath). Brackets and whitespaces in each data row are removed.

Example **.xlsx file**:

Coding region change
ENST00000413998:c.[1519G>A]
ENST00000413998:c.[1520T>C]; ENST00000372470:c.[1520T>C]
ENST00000413998:c.[1521C>T]; ENST00000372470:c.[1521C>T]
ENST00000413998:c.[1522C>T]; ENST00000372470:c.[1522C>T]
ENST00000413998:c.[1523T>A]; ENST00000372470:c.[1523T>A]
ENST00000413998:c.[1523T>C]; ENST00000372470:c.[1523T>C]

Information must be given in this way

AMPLICON DESIGN

Additionally, the user can load a file containing information about amplicon design. This is only used for calculation of clones if the user ticks the "Infer Relationship(s)" checkbox. This comes from the assumption that positions can be covered multiple but different times with overlapping amplicons and paired end reads.

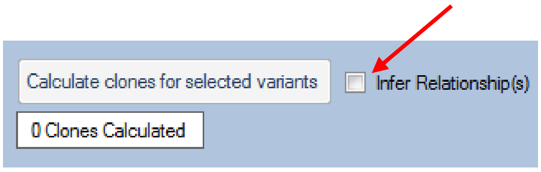

The user has to provide a CSV file that includes the ranges of the amplicons. The file must follow the structure "start;end;start;end" (covered regions of the paired end amplicons) and must not contain any header, e.g.:

1	20	21	40
1	20	41	60
1	20	61	80
21	40	41	60
21	40	61	80
41	60	61	80

amplicon design example

Export

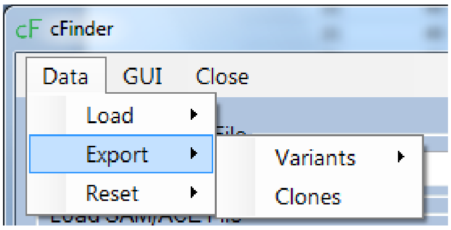

Using this menu section, the user is able to export variants (shown in the variants output window) or clones (shown in the sequence clones output window) with whole information that is given.

Export variants

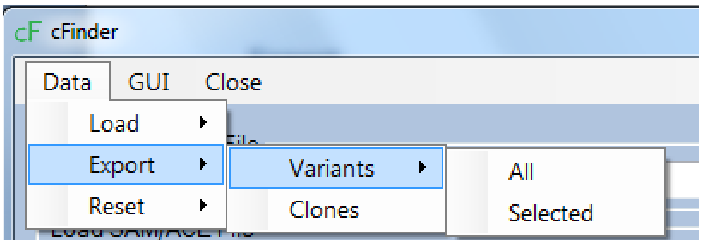

These variants can be loaded again e.g. for another sample or monitoring purposes (please see section "*Menu – Load – User-Defined Variants File*").

Export

- *all*
  If this menu item is clicked, all variants of the variants output window are exported to a file (a dialog window appears where a location and file path can be chosen).
- *selected*
  Just the selected variants are exported to a file.

Export clones

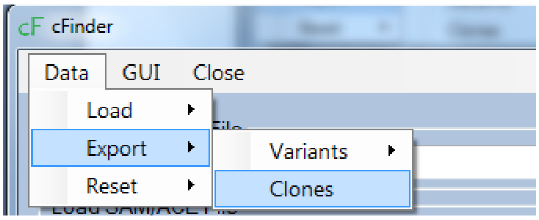

For exporting clones, they have to be calculated first (*please see section "Sequence Clone Calculation").* All calculated clones are exported to a CSV file containing the information as shown in the sequence clone output window.

	A	B	C	D	E
1	Clone	Coding Region Change	Mutation AA	Counter Clor	Percentage
2	T/C | A/- |	c.678T>C | c.1073delA	p.?226 | p.N:	1	0,1333
3	T/C | -/C |	c.678T>C | c.887_888in	p.?226 | p.P2	1	0,1242
4	T/C | A/-	c.681T>C | c.1073delA	p.G227G | p.	2	0,2465
5	T/C	c.681T>C	p.G227G	1	0,1435

Export: clones

Reset

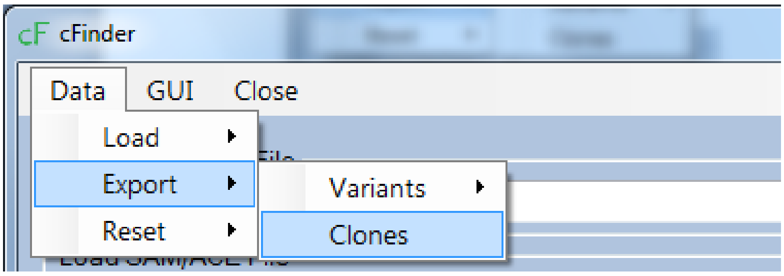

There are three options of reset by menu selection for loading new data.
See also section “*Reset automatically*”.

- Annotations File
  If an annotations file has been loaded before and the user wants to annotate an already parsed sequencing file in a different way, the annotation information can be reset with this option. The variant data remains saved and will just be annotated in another way than before. Before reading the new annotation file, all output windows are cleared and all filters removed (default filters are restored).
- Mutations File and Output
  This option resets the sample file. The annotation data, if existing, is preserved and used again for annotating the new calculated variants. Before reading the new SAM or ACE file, all output windows are cleared and all filters (except the default filters) removed.
- All
  Clears all data structures, output windows and read in data.

Close

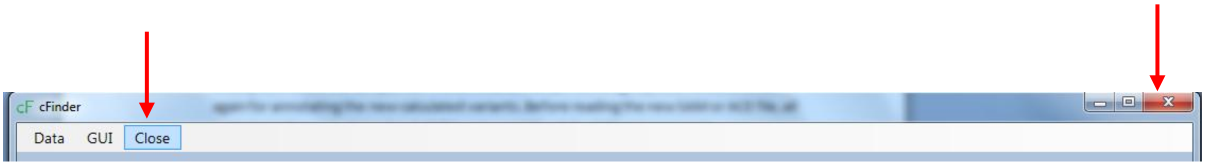

The “Close” menu item and the usual closing operation of the graphical user interface both close the application. The logfile is written at this point.

Reset automatically

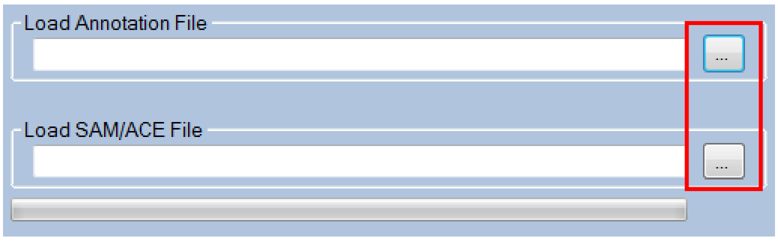

If the user wants to reset only the annotation data or the sequences file, it could also be done by pushing one of the “load file” buttons (red mark).

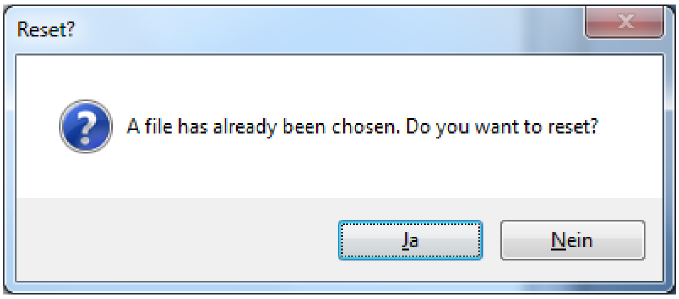

If data has already been loaded from a file, a message box appears to inform and ask the user if another file has to be loaded.

Annotation File

If annotation data has been loaded before, the annotation information can be reset with this option. The variant data will not be changed and just annotated in a different way.

Sequences File

Reset the SAM/ACE file for new variant calculation. The annotation data, if exists, will not be changed and will be used for annotating the new calculated variants.

Logger

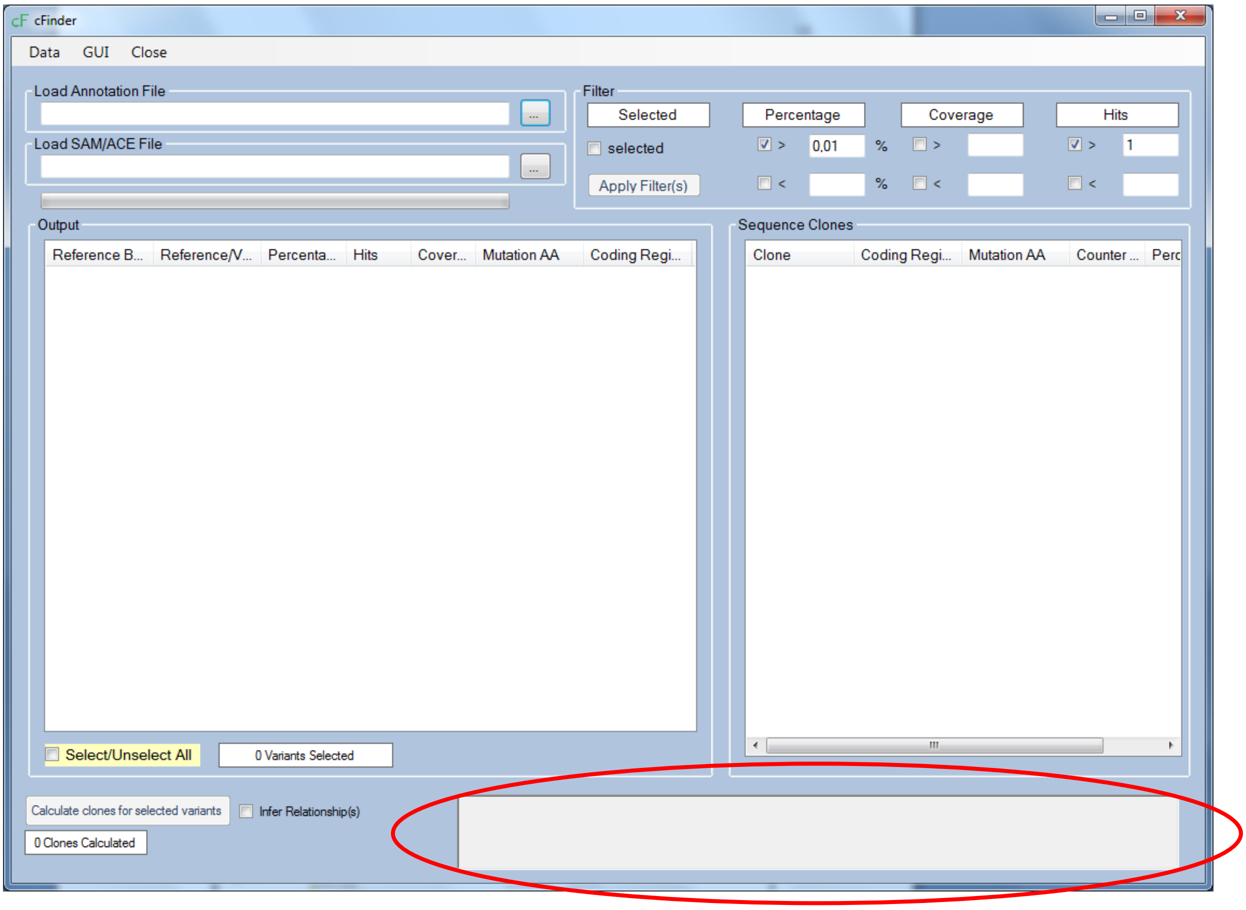

The logger reports all events that are caused by a user's activity. So the logger is used for creating an error log file that reports normal activities but also error messages. So each step is documented and reproducible if an error occurs.

The memory location of the user's activity log can be changed by the user himself. Therefore, the setup file for user settings has to be changed. This file is called *settings.xml* and is located in the project file folder. This file looks like this:

```
<?xml version="1.0" encoding="UTF-8"?>
<!DOCTYPE UserSettings SYSTEM "userSettings.dtd">

<UserSettings>
    <logfile>\\bz-srv-file03\abteilungen$\Genomics\Bioinformatik</logfile>
</UserSettings>
```

There you see that in the *logfile* section a file path for saving the user activity is already entered. This file path can be changed doing the following steps:

1. Open the file folder where the activity file should be created automatically.
2. Click next to the directory path in the directory window:

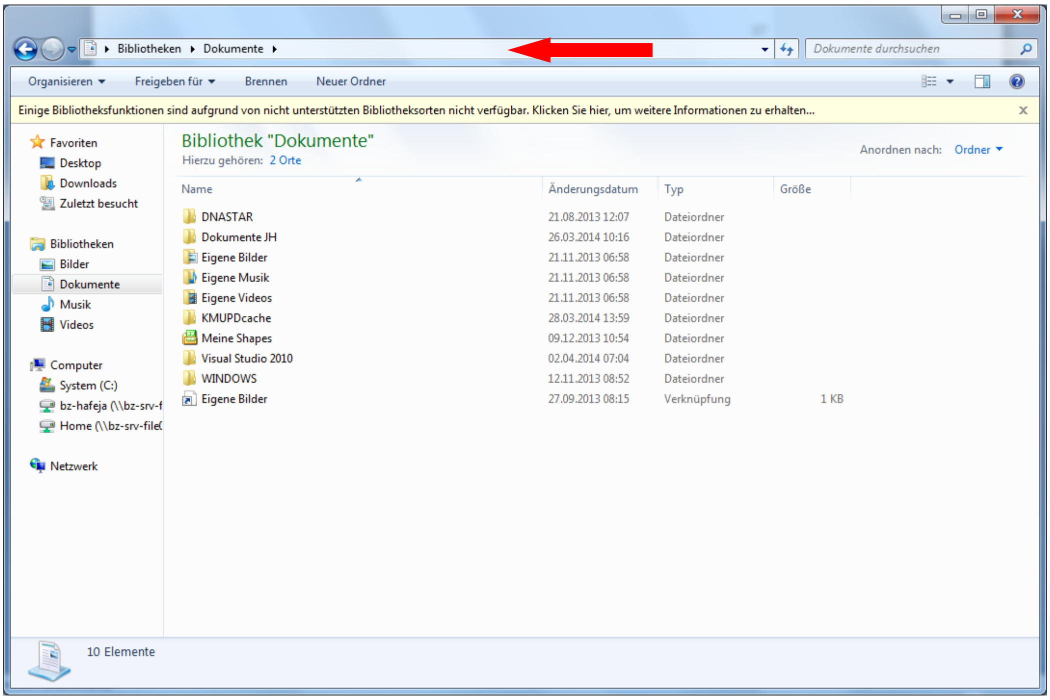

Then the path of the directories will be displayed like this:

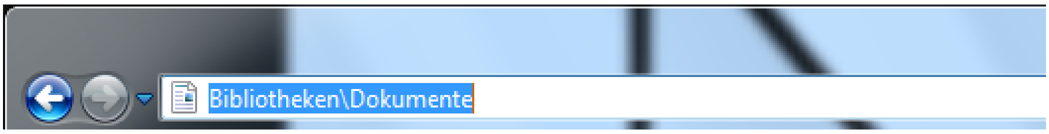

3. Copy this file path from the top of the directory and paste it between the logfile tags in the *settings.xml* document:

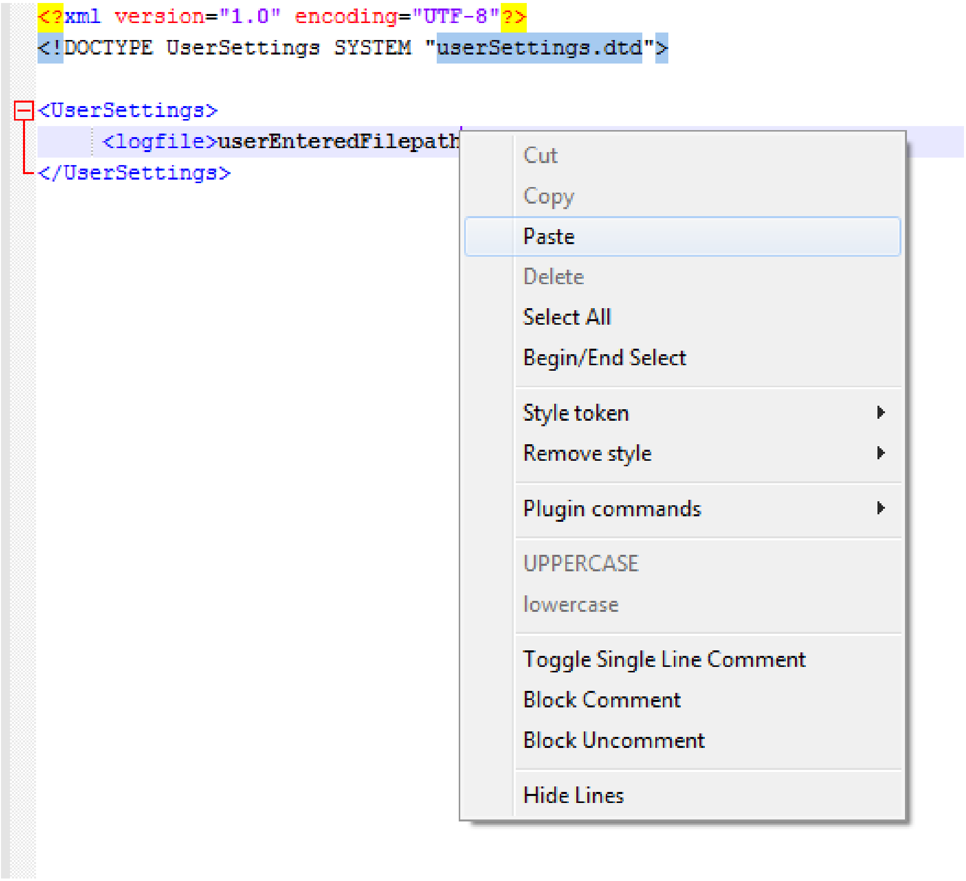

4. Save the document.
5. Now run the application.

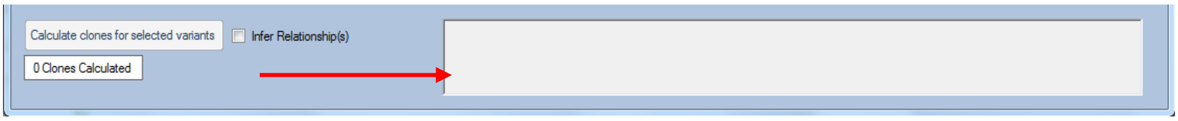

Each important entry that is reported by the logger is also shown at the GUI (red arrow). So the user can follow the progress and trace his actions.

Features

Menu: GUI - Change color

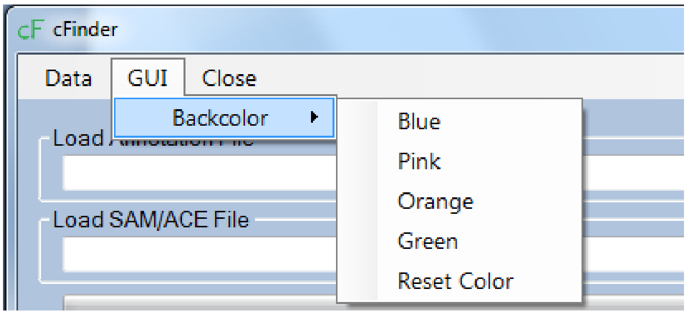

Because the software is very user-friendly, the user is able to change the color of the GUI, or to reset it to the standard color.

Blue

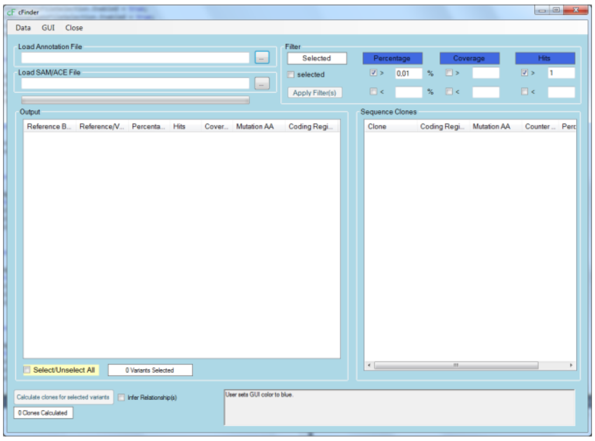

Pink

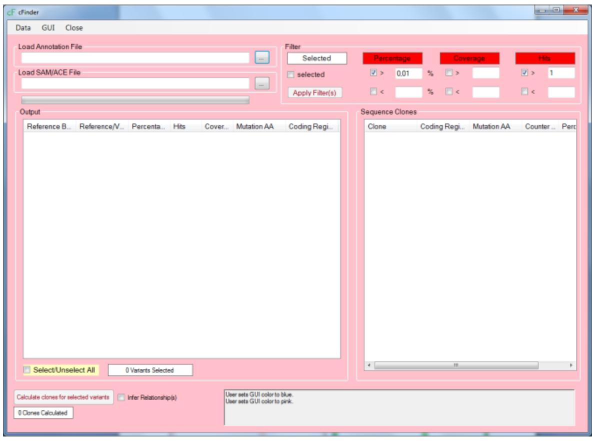

Orange

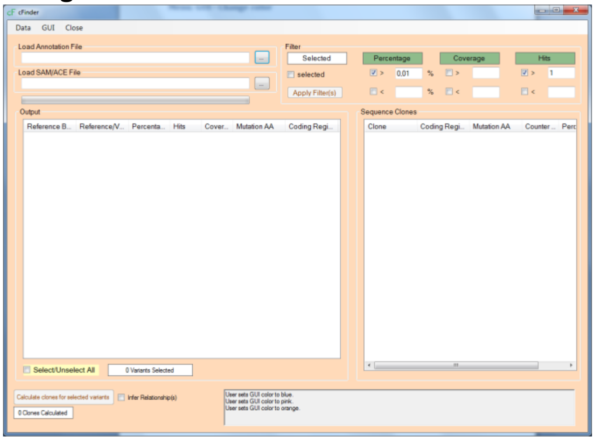

Green

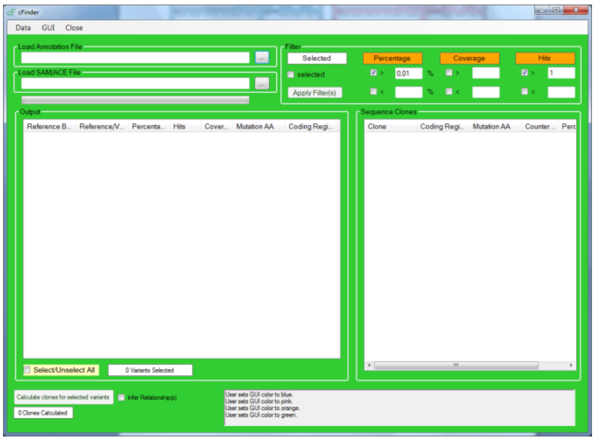

Change window size

The user is able to resize both output windows with a click between them for shifting the bar to the left or to the right. Furthermore, the window is resizable too.

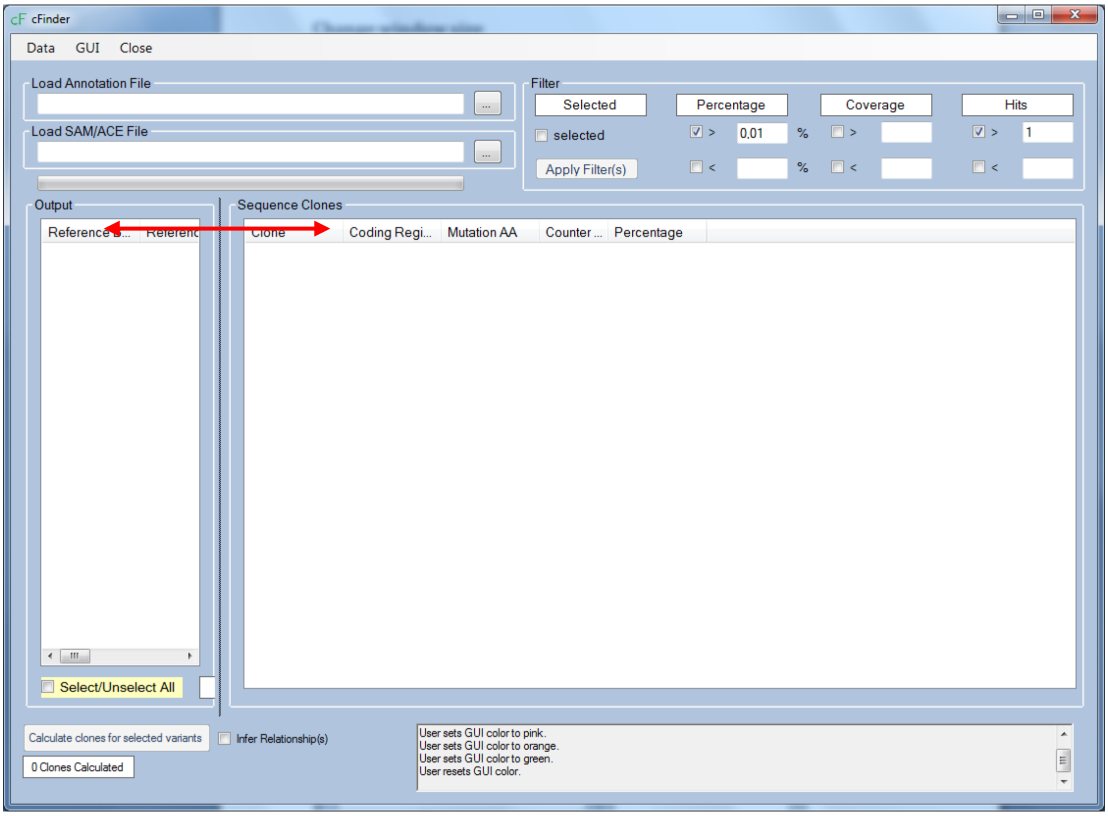

Resizing of the two output windows

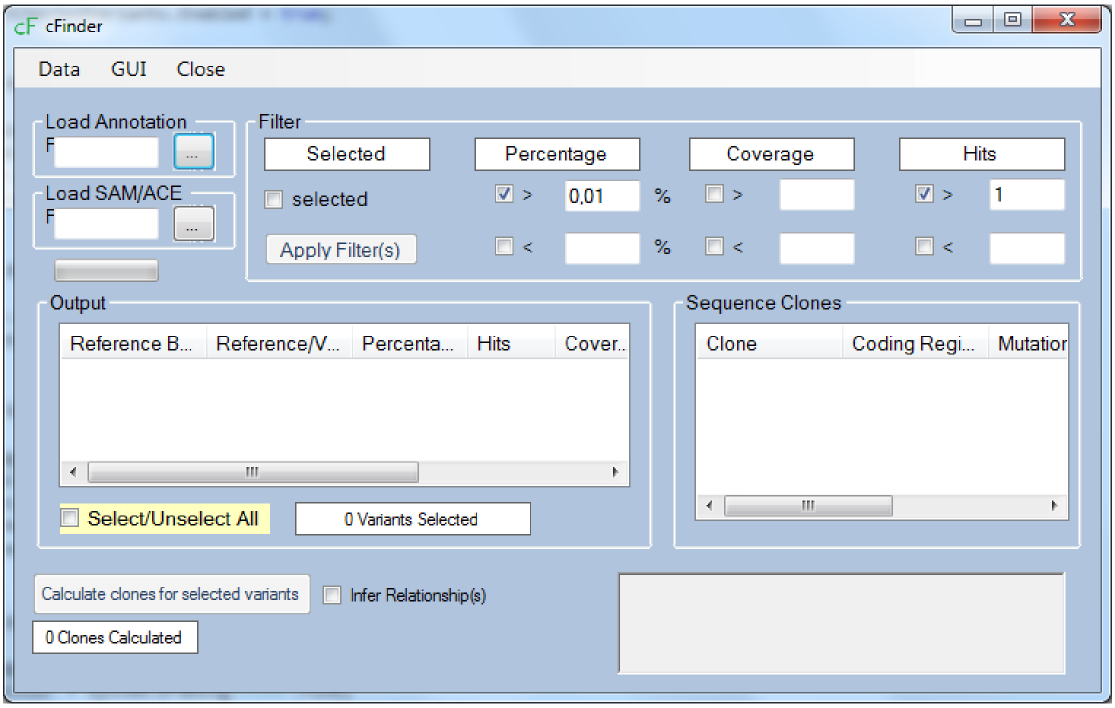

Resizing application window
